# A peripheral lipid sensor GPR120 remotely contributes to suppression of PGD_2_-microglia-provoked neuroinflammation and neurodegeneration in the mouse hippocampus

**DOI:** 10.1186/s12974-021-02361-2

**Published:** 2021-12-27

**Authors:** Kensuke Iwasa, Shinji Yamamoto, Kota Yamashina, Nan Yagishita-kyo, Kei Maruyama, Takeo Awaji, Yoshinori Takei, Akira Hirasawa, Keisuke Yoshikawa

**Affiliations:** 1 Department of Pharmacology, Faculty of Medicine, Saitama Medical University, 38 Moro-hongo, Moroyama-machi, Iruma-gun, Saitama, 350-0495 Japan; 2 Department of Translational Research and Cellular Therapeutics, School of Medicine, Faculty of Medicine, Toho University, 5-21-16 Omori-Nishi, Ota-ku, Tokyo, 143-8540 Japan; 3 Department of Genomic Drug Discovery Science, Graduate School of Pharmaceutical Sciences, Kyoto University, 46-29 Yoshida-Shimo-Adachi-cho, Sakyo-ku, Kyoto, 606-8501 Japan; 4 Institute for Integrated Medical Sciences, Tokyo Women’s Medical University, 8-1 Kawada-cho, Shinjuku-ku, Tokyo, 162-8666 Japan

**Keywords:** Prostaglandin, G-protein-coupled receptor 120 (GPR120), Neuroinflammation

## Abstract

**Background:**

Neuroinflammation is a key pathological component of neurodegenerative disease and is characterized by microglial activation and the secretion of proinflammatory mediators. We previously reported that a surge in prostaglandin D_2_ (PGD_2_) production and PGD_2_-induced microglial activation could provoke neuroinflammation. We also reported that a lipid sensor GPR120 (free fatty acid receptor 4), which is expressed in intestine, could be activated by polyunsaturated fatty acids (PUFA), thereby mediating secretion of glucagon-like peptide-1 (GLP-1). Dysfunction of GPR120 results in obesity in both mice and humans.

**Methods:**

To reveal the relationship between PGD_2_-microglia-provoked neuroinflammation and intestinal PUFA/GPR120 signaling, we investigated neuroinflammation and neuronal function with gene and protein expression, histological, and behavioral analysis in GPR120 knockout (KO) mice.

**Results:**

In the current study, we discovered notable neuroinflammation (increased PGD_2_ production and microglial activation) and neurodegeneration (declines in neurogenesis, hippocampal volume, and cognitive function) in GPR120 KO mice. We also found that Hematopoietic–prostaglandin D synthase (H-PGDS) was expressed in microglia, microglia were activated by PGD_2_, H-PGDS expression was upregulated in GPR120 KO hippocampus, and inhibition of PGD_2_ production attenuated this neuroinflammation. GPR120 KO mice exhibited reduced intestinal, plasma, and intracerebral GLP-1 contents. Peripheral administration of a GLP-1 analogue, liraglutide, reduced PGD_2_-microglia-provoked neuroinflammation and further neurodegeneration in GPR120 KO mice.

**Conclusions:**

Our results suggest that neurological phenotypes in GPR120 KO mice are probably caused by dysfunction of intestinal GPR120. These observations raise the possibility that intestinal GLP-1 secretion, stimulated by intestinal GPR120, may remotely contributed to suppress PGD_2_-microglia-provoked neuroinflammation in the hippocampus.

**Supplementary Information:**

The online version contains supplementary material available at 10.1186/s12974-021-02361-2.

## Background

Prostaglandins (PGs) are arachidonic acid-derived lipid mediators that exert diverse biological activities through their cognate G-protein-coupled receptors (GPCRs) [1]. PGD_2_, one of the most abundant PGs in the brain, is generated by two PGD synthases (PGDSs), hematopoietic- and lipocalin-type PGDS (H-PGDS and L-PGDS, respectively), and signals through two distinct GPCRs, DP1 and DP2 (CRTH2) [2, 3]. Neuroinflammation, as typified by microglial activation and secretion of proinflammatory mediators, is a major contributor to neurodegeneration [4]. Neurodegeneration refers to the loss of neuronal function caused by atrophy (reduced brain volume), neuronal death, or impaired neurogenesis, which are hallmarks of neurodegenerative disease [4–6]. Activated microglia secrete proinflammatory mediators, such as PGs, and mediate neuronal injury, which exacerbates neurodegeneration [7]. We previously reported that a surge in PGD_2_ production and PGD_2_-induced microglial activation provoke neuroinflammation and further neurodegeneration in excitotoxic hippocampal lesion [8–10]. Thus, a surge in PGD_2_ production and microglial activation are closely connected with neuroinflammation and neurodegeneration in the neurological deficit.

We also previously reported that GPR120 (free fatty acid receptor 4) is expressed in intestinal enteroendocrine cells [11] and is a receptor of polyunsaturated fatty acids (PUFA), such as α-linolenic acid (ALA), eicosapentaenoic acid (EPA), and docosahexaenoic acid (DHA) [11, 12]. GPR120 senses PUFA and mediates the secretion of a gut-derived incretin hormone, glucagon-like peptide-1 (GLP-1), which promotes insulin secretion [11, 13]. Furthermore, dysfunction of GPR120 results in dietary obesity in both mice and humans [13]. Recently, GLP-1 biological activity has become the basis for incretin-based therapies for type 2 diabetes mellitus, including liraglutide, an agonist of the GLP-1 receptor [14, 15]. In addition to the peripheral level of GLP-1, GLP-1 readily crosses the blood–brain barrier (BBB) and stimulates the GLP-1 receptor expressed in the brain [16, 17]. Potentiation of intracerebral GLP-1 bioactivity has been shown to increase neuronal activity, promote neuronal growth, and have neuroprotective properties [18, 19].

In the current study, we evaluated neuroinflammation and neuronal function in GPR120 KO mice to reveal the relationship between PGD_2_-microglia-provoked neuroinflammation and intestinal PUFA/GPR120 signaling. We demonstrated that GPR120 KO mice exhibited declines in neurogenesis, hippocampal volume, and cognitive function, which are manifestations of neurodegeneration. The neurodegeneration observed in GPR120 KO mice was caused by consistent PGD_2_-microglia-provoked neuroinflammation in the hippocampus. Importantly, GPR120 mRNA was detected in the intestinal tissues and GPR120 KO mice exhibited reduced intestinal, plasma, and intracerebral GLP-1 contents. Peripheral administration of a GLP-1 analogue, liraglutide, prevented PGD_2_-microglia-provoked neuroinflammation and further neurodegeneration in GPR120 KO mice. These observations raise the possibility that intestinal GLP-1 secretion, stimulated by GPR120, remotely contributed to hippocampal homeostasis via suppression of PGD_2_-microglia-provoked neuroinflammation.

## Methods

### Animal procedures

Mice lacking GPR120 are described previously [13]. The established mixed C57BL/6/129 background GPR120 KO mice were backcrossed into the C57BL/6 J background using a marker-assisted breeding approach [20]. Genotyping of the GPR120 KO mice was performed using the primers, Forward: 5′-aagtcaatcgcacccacttc-3′ Reverse: 5′-caagctcagcgtaagcctct-3′. Male WT C57BL/6 J (Tokyo Laboratory Animals Science, Tokyo, Japan) and GPR120 KO mice were maintained on a 12 h/12 h light/dark cycle with free access to a powdered diet (CLEA Japan, Tokyo, Japan) and tap water. In our previous report [13], when GPR120 KO mice were fed a high-fat diet, their average body weight was higher than that of WT mice fed a high-fat diet. By contrast, body weight did not differ significantly between 16-week-old WT and GPR120 KO mice fed a normal diet. In this study, we used 16-week-old male WT and GPR120 KO mice fed a normal diet. No difference in body weight between GPR120 KO (25.9 ± 0.75 g) and WT (25.7 ± 0.58 g) mice was observed in any of our experiments. All animal studies were approved by the Institutional Animal Care and Use Committee (3186, 3196) and DNA experiment Safety Committee of Saitama Medical University (1530).

### Chemicals

Kainic acid (KA, 78050, Cayman Chemicals, Ann Arbor, MI), indomethacin (IND, 19233-51, Nacalai tesque, Tokyo, Japan), liraglutide (2499410G1021, Novo Nordisk, Bagsværd, Danmark), and sitagliptin phosphate monohydrate (SPM, A4036, ApexBio, Boston, MA, USA) were used for animal treatments. Fluoro Jade C (FJC, TR-100-FJ, Biosensis, CA) was used for staining of degenerating neurons. Pre-stained Protein Marker (02525-35, Nacalai tesque, Tokyo, Japan) was used for Western blots. MK-0524 (DP1 antagonist, 1480, Axon MEDCHEM, Groningen, Netherlands), OC000459 (DP2 antagonist, 1913, Axon MEDCHEM, Groningen, Netherlands) were used as DP selective inhibitors.

### Pharmacological treatments

For IND studies, 5-week-old mice were placed on a powdered diet containing 0.01% IND for a total period of 11 weeks. For liraglutide studies, 5-week-old mice were inserted subcutaneously an Alzet osmotic pump (Muromachi, Tokyo, Japan) filled saline dissolved liraglutide in the abdomen. The pumps delivered saline or 200 mg/kg of liraglutide per day for 11 weeks. SPM (50 mg/kg per day) was orally administrated to 15-week-old mice for a week. The dose of liraglutide and SPM was based on previous reports [21, 22].

### Antibodies

We used the following antibodies: anti-Ionized calcium binding adapter molecule 1 (Iba-1, Western blot (WB); 1: 1000, Immunohistochemistry (IHC); 1:500, 019-19741, Wako, Osaka, Japan), anti-cyclooxygenase-1 (COX-1, WB; 1: 200, sc-19998, Santa Cruz Biotechnology, CA), anti-COX-2 (WB; 1: 100, sc-376861, Santa Cruz Biotechnology, CA), anti-L-PGDS (WB; 1: 500, PA1-46023, Thermo Fisher Scientific, Tokyo, Japan), anti-H-PGDS (WB; 1: 1000, PA5-24347, Thermo Fisher Scientific, Tokyo, Japan), anti-doublecortin (DCX, WB; 1: 1000, IHC; 1: 2000, ab18723, Abcam, Cambridge, MA), anti-Ki67 (IHC; 1: 100, NB500-170, Novus Biologicals, Inc., Littleton, CO), anti-superoxide dismutase 2 (SOD2, WB; 1: 1000, 13194, Cell Signaling Technology, Beverly, MA), anti-14-3-3ς (WB; 1: 1000, 7413, Cell Signaling Technology, Beverly, MA), anti-synaptophysin (WB; 1: 20000, ab32127, Abcam, Cambridge, MA), anti-postsynaptic density protein 95 (PSD95, WB; 1: 250, 610495, BD Biosciences, San Diego, CA), anti-Nuclear factor erythroid 2-related factor 2 (Nrf2, WB; 1: 500, Proteintech, Chicago, MA), anti-α-tubulin (WB; 1: 4000, T5168, Sigma-Aldrich, Deisen-hofen, Germany), and anti-GAPDH (WB; 1: 1000, ABS16, Millipore, Billerica, MA).

### Histology

Mice were intracardially perfused with 4% paraformaldehyde in phosphate buffered saline (PBS) after isoflurane (099-06571, Wako, Japan) anesthesia. Brains were removed and postfixed overnight in 4% paraformaldehyde in PBS and subsequently cryoprotected in 30% sucrose solution in PBS, snap frozen and stored at − 80 °C until required. Coronal brain sections (25 μm thick) were cut on a cryostat (LEICA CM1900, Wetzlar, Germany) and mounted on gelatin-coated glass slides. Nissl staining was performed according to standard protocols. Sections were cover slipped using Poly-Mount (Polysciences Inc. Boston, MA). For FJC staining, KA (10 mg/kg, dissolved in saline) were injected intraperitoneally into the WT mice. Mice were intracardially perfused with 4% paraformaldehyde in PBS after 24 h. FJC staining was performed according to the manufacturer's instruction [10]. Slides were incubated in sodium hydroxide for 5 min, then washed with 70% EtOH followed by distilled water. Slides were then incubated in potassium permanganate for 10 min. Next, slides were washed with distilled water and moved to low-light for staining with FJC and 4, 6-diamidino-2-phenylindole (DAPI) for 15 min. Slides were rinsed with distilled water, and cleared by brief immersion in xylenes. Slides were then coverslipped using DPX (Merck KGaA, Darmstadt*,* Germany).

For immunohistochemistry, sections were incubated for 1 h in a blocking buffer (PBS 5% BSA, 0.1% Polyoxyethylene Sorbitan Monolaurate) and incubated with the primary antibody (anti-Ki67), at 4 °C overnight, followed by incubation for 1 h with secondary anti-rabbit IgG antibody conjugated with horse-radish peroxidase (Envision + System, Dako, Glostrup, Denmark). Ki67 positive cells in dentate gyrus were counted. For immunofluorescence, sections were incubated with the primary antibodies (anti-Iba-1 and anti-DCX) after blocking, at 4 °C overnight, followed by incubation for 1 h with secondary antibody (Cy3-conjugated AffiniPure goat anti-Rabbit IgG; 1: 500, Jackson ImmunoReseach, inc. PA) in the dark at 25 °C. Sections were cover slipped using DPX. Sections were photographed at 40 × magnification, and images were captures using a KEYENCE BZ-X710 microscope (Keyence Corporation, Osaka, Japan). Iba-1 positive cells in the hippocampus, CA1, and CA3, and DCX positive cells in the dentate gyrus were counted, and densities (counts/mm^2^) were calculated.

### Measurements of hippocampal and cortical volume

Serial coronal brain slices were cut at a thickness of 25 μm using a cryostat. H&E staining was performed according to standard protocols. Areas of the left hippocampus and cortex (primary somatosensory cortex, motor cortex, and insular cortex) were measured in every 100 μm that contained whole hippocampus and cortex using a KEYENCE BZ-X710 microscope. These areas (mm^2^) × 0.1 (mm) from all sections were summed and recorded as a unilateral hippocampal and cortical volume (mm^3^) [9]. Relative values of hippocampal and cortical volume were represented as a percentage of the average volume of the same structures in the control mice.

### Western blotting

Brain tissues and primary cell cultures were homogenized on ice in RIPA buffer [50 mM Tris–HCl pH 8.0, 150 mM NaCl, 5 mM ethylenediaminetetraacetic acid (EDTA), 1% NP-40, 0.1% sodium dodecyl sulfate (SDS), 0.5% deoxycholate (DOC)] buffer containing 1:1000 dilution of a protease inhibitor cocktail (CalBiochem, San Diego, CA, USA) with a tissue homogenizer (Brinkmann Instruments, Westbury, NY, USA). Protein concentrations were determined using a BCA protein assay kit (Nacalai tesque, Tokyo, Japan). 10 μg protein/lane of lysates was subjected to SDS–polyacrylamide gel electrophoresis and transferred to nitrocellulose membranes (Bio-Rad, Redmond, WA). After blocking with 5% skim milk (MEGMILK SNOW BRAND Co Ltd, Tokyo, Japan) in PBS containing 0.05% Tween 20 (Polyoxyethylene Sorbitan Monolaurate, Nacalai tesque, Tokyo, Japan) (PBS-T), the membranes were incubated with the primary antibodies overnight, followed by incubation with horseradish peroxidase-conjugated secondary antibodies (Cell Signaling Technology, Beverly, MA) and washing with PBS-T three times. The membranes were treated with reagent for exposure (Chemi-Lumi One Super, Nacalai tesque, Tokyo, Japan; ImmunoStar LD, Wako, Japan). Image of the membranes was captured using a C-DiGit blot scanner (LI-COR, Lincoln, NE) and subjected to ImageJ analysis. Each membrane was probed with only 1 antibody, with α-tubulin or GAPDH used as a loading control. A pre-stained molecular weight marker confirmed the expected size of the target proteins.

### ELISA analysis

To determine PGD_2_ concentration in the WT and GPR120 KO hippocampus, KA (10 mg/kg, dissolved in saline) were injected intraperitoneally into the WT mice. After 30 min, mice euthanized, and hippocampi were collected. Total lipids were extracted using n-hexane/2-propanol (3:2, by vol, HIP). The HIP (5 μl/mg tissue) was added to hippocampal tissue. Samples were homogenized at maximum speed. The homogenate was centrifuged at 1500 × *g* for 10 min at room temperature. The supernatant fraction was decanted and being dried down using an integrated SpeedVac® concentrator (SPD111V, Thermo Scientific, Rockford, IL, USA). The fraction was diluted 500 μl in assay buffer. The PGD_2_, PGE_2_, and PGF_2α_ concentration was assayed with each EIA kit (Cayman Chemicals, Ann Arbor, MI).

The WT and GPR120 KO mice were fasted overnight. For plasma GLP-1 quantification, blood samples were collected in test tubes containing a sitagliptin (100 μM) and then centrifuged for 20 min at 1200 × *g* at 4 °C. Intestinal and intracerebral GLP-1 was extracted according to the method by Cani et al*.* [23] and McClean et al*.* [24], respectively. The acid ethanol (75% ethanol + 0.15 mol/L hydrochloric acid) was added to intestinal and brain tissue. Samples were homogenized at maximum speed and placed at 4 °C for 24 h. The homogenate was centrifuged at 5000 × *g* for 20 min at 4 °C. The supernatant fraction was decanted and being dried down using an integrated SpeedVac® concentrator. The active GLP-1 concentration was assayed with Active GLP-1 ELISA Kit (FUJIFILM, Gunma, Japan). Results were measured in a Benchmark Microplate Reader (Bio-Rad, Redmond, WA).

### RNA extraction and quantitative real-time PCR (Q-PCR)

Tissue samples and neuronal and glial primary cell cultures were processed for RNA extraction using ISOGEN (NIPPON GENE, Tokyo, Japan) following the manufacturer's instructions. RNA was reverse transcribed using PrimeScript RT reagent kit (TAKARA BIO INC, Shiga, Japan) reverse transcriptase. Q-PCR was performed using the Quant Studio 12 K Flex (Applied Biosystems, CA). The following primer sequences were used: Phosphoglycerate kinase 1 (*PGK1*; Forward: 5′-tgctgttccaagcatcaaa-3′ Reverse: 5′-gcatcttttcccttcccttc-3′); *GPR120* (Forward: 5′-gtcgtctgccacctgctctt-3′ Reverse: 5′-tttctcctatgcggttgggc-3′); *NeuN* (Forward: 5′-agcagcccaaacgactacat-3′ Reverse: 5′-acaagagagtggtgggaacg-3′); *GFAP* (Forward: 5′-gcttcctggaacagcaaaac-3′ Reverse: 5′-cggcgatagtcgttagcttc-3′); *Iba-1* (Forward: 5′-gaagcgaatgctggagaaac-3′ Reverse: 5′-gaccagttggcctcttgtgt-3′); *SOD2* (Forward: 5′-ggccaagggagatgttacaa-3′ Reverse: 5′-gaaccttggactcccacaga-3′); *14–3-3ς* Forward: 5′-cccattcgtttaggtcttgc-3′ Reverse: 5′-cctgcagcgcttctttattc-3′); *COX-1* (Forward: 5′-cagtgcctcaaccccatagt-3′ Reverse: 5′-gtggctatttcctgcagctc-3′); *COX-2* (Forward: 5′-ccccaaagatagcatctgga-3′ Reverse: 5′-gctgtacaagcatggcaaa-3′); *L-PGDS* (Forward: 5′-catagttggccaccact-3′ Reverse: 5′-tccgggagaagaaagctgta-3′); *H-PGDS* (Forward: 5′-cgaggtgcttgatgtgtgag-3′ Reverse: 5′-tgttttggaggtggaaggac-3′); *GLP-1 receptor* (Forward: 5′-ccaggttccttcgtgaatgt-3′ Reverse: 5′-caaggcggagaaagaaagtg); Tumor necrosis factor α (*TNFα*, Forward: 5′-gcctcttctcattcctgctt-3′ Reverse: 5′-cacttggtggtttgctacga-3′); Interleukin-1β (*IL-1β* Forward: 5′-gaccttccaggtgaggaca-3′ Reverse: 5′-aggccacaggtattttgtcg-3′); *IL-6* (Forward: 5′-aacgatgatgcacttgcaga-3′ Reverse: 5′-ggaaattggggtaggaagga-3′).

### Behavioral tests

The Y-maze apparatus (Hazai-ya, Tokyo, Japan) was a 3-arm radial maze with equal angles between all arms (8 cm width) and a bottom with 40 cm (length) and 15 cm height. Mice were tested individually by placing them in an arm of the maze and allowing them to move freely throughout the 3 different arms for 10 min. The sequence and entries into each arm were recorded. An alternation was determined from successive consecutive entries into the 3 different arms on overlapping triads in which all arms were represented. For example, ACBABCABAB, a sequence of entries to the 3 arms A, B, or C, would generate 5 ‘successful’ alternations, ACB, CBA, ABC, BCA, and CAB; the total number of possible alternations corresponded to the number of the total arm entries minus 2 (in this example, the total number would equal 8). The percentage alternation was calculated as (the number of ‘successful’ alternations divided by the number of the total arm entries minus 2) × 100. We analyzed the percentage alternation and the total number of arm entries.

Morris water maze consisted of circular pool (diameter, 150 cm; height, 40 cm) was divided into four quadrants (north, east, west, and south) and at the center of the north quadrant, a platform was placed. The geometric shapes were pasted at the walls for visual cues. A 10 cm transparent platform was placed 1 cm beneath the surface of the water and 40 cm from the wall in the South–West quadrant of the pool. Mice were placed in a quadrant and given time to find the platform in 90 s during the first 5 days (escape latency). If the animal did not find the platform at the set time, its handler directed to the platform in training. The next 5 days, the platform was removed, the amount of time the mice spend in proximity to its former location is gauged (known as a probe trial) to assess memory. The mice were allowed 300 s to swim to evaluate their reference memory (cross-platform time). Mice were video tracked and analyzed behavioral parameters.

### Cell cultures

Primary cell cultures were separately isolated following the method [25]. Primary neurons were prepared from cerebral cortex of embryonic day 18 mouse embryo. Brains were stripped of meninges and dissected from diencephalon, were dispersed and incubated at 37 °C in Hank’s balanced salt solution (HBSS) containing 0.25% trypsin (Life Technologies, CA) and 0.001% DNase I (Roche Diagnostics, Mannheim, Germany). After inhibiting the trypsin with fetal bovine serum, the suspension was again disrupted with a pipette and filtered through a 70 μm nylon mesh (BD Falcon, MA). The filtered cell suspension was placed in poly-l-lysine-coated 75 cm^2^ flasks and kept at 37 °C in a humidified incubator with 5% CO_2_ in air. Neuronal cells were cultured in Neurobasal Medium (Life Technologies, CA) with B27 supplement. After 1 day, the medium was replaced with Neurobasal Medium. The culture medium was subsequently changed twice a week. Cells were harvested after 14 days in vitro.

Primary mixed glial cultures (astrocytes and microglia) were prepared from forebrains of postnatal 2-day-old mice using a differential detachment method [26, 27]. Briefly, forebrains free of meninges were digested with HBSS containing 0.25% trypsin and 0.001% DNase I and triturated with DMEM containing 10% heat-inactivated fetal bovine serum and 1% penicillin–streptomycin. Dissociated cells were plated in poly-l-lysine-coated 75 cm^2^ flasks. The culture medium was changed twice a week. Astrocytes were detached from the 75 cm^2^ flasks by trypsinization. Individual glial cells were used for the experiments. After 14 days in vitro, when cultures reached a confluence, microglia were isolated by shaking the mixed glia-containing flasks for 1 h at 200 rpm and plated with 500,000 cells/well in 6-well plates. After resting for 24 h, microglia were stimulated with 100 ng/ml Lipopolysaccharide (LPS, Sigma, Deisen-hofen, Germany) 1 h after pretreatment with 10 ng/ml liraglutide. In the PGD_2_ addition experiment, microglia were incubated with the medium containing rat recombinant GM-CSF (20 ng/mL, Pepro Tech, London, UK) after plated with 500,000 cells/well. After resting for 24 h, cells were stimulated with 1 μM PGD_2_ (Cayman CHEMICAL, Ann Arbor, MI) 1 h after pretreatment with 1μm MK-0524, 1 μM OC000459.

### Statistics

Two-sample comparisons were carried out using a student’s *t* test. Multiple comparisons were performed by one-way ANOVA followed by Newman–Keuls post-hoc test or two-way ANOVA followed by post-hoc Tukey test. All data were analyzed using Graph Pad Prism Ver. 5.01 (Graph Pad Software, Inc., San Diego, CA) and expressed as mean ± SEM. *p* values < 0.05 were considered statistically significant.

## Results

### Tissue distribution analysis of GPR120 mRNA and declines in hippocampal volume, neurogenesis, and cognitive function observed in GPR120 KO mice

**Fig. 1:**
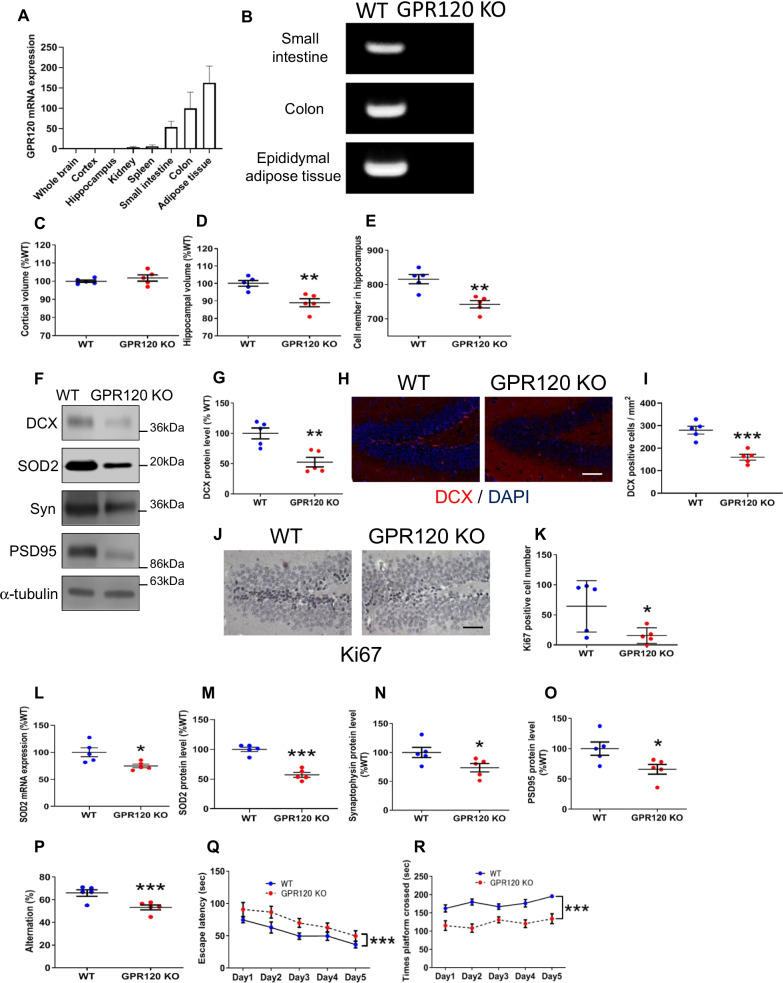
Declines in hippocampal volume, neurogenesis, and cognitive function observed in GPR120 KO mice. The level of GPR120 mRNA (**A**) relative to PGK1 in WT mice tissues, as determined by real-time PCR. Genotyping of GPR120 gene in small intestine, colon, and epididymal adipose tissue (**B**). Cortical (**C**) and Hippocampal (**D**) volumes of WT and GPR120 KO mice. Nissl staining and pyramidal cell counts of hippocampus (**E**). Scale bar = 80 μm. The protein level determined by western blot analysis (**F**). The protein level of DCX in the hippocampus (**G**). The immunofluorescence of DCX (**H**) and DCX-positive cell count in the dentate gyrus (**I**). Scale bar = 80 μm. Ki67 staining (**J**) and the number of Ki67-positive nuclei in the dentate gyrus (**K**). Scale bar = 80 μm. The level of SOD2 mRNA (**L**) and protein (**M**) expression in the hippocampus. The level of synaptophysin (Syn) (**N**) and PSD95 (**O**) protein in the hippocampus. Learning and memory performance were evaluated using the Y-maze (**P**). Data are presented as the mean ± SEM, *n* = 5 per group. Statistical analysis was performed using a student’s *t* test (*p < 0.05; **p < 0.01; ***p < 0.001 vs. WT). Morris water maze test: Escape latency (**Q**) and Time platform crossed (**R**). Data are mean ± SEM, *n* = 10 per group. Statistical analysis was performed using two-way ANOVA followed by post-hoc Tukey test (***p < 0.001 vs. WT)

Tissue distribution analyses in the WT mice showed abundant expression of GPR120 mRNA in the small intestine, colon, and adipose tissues (Fig. 1A). We could not detect GPR120 mRNA in the whole brain, hippocampus, or cortex. Genotyping analysis confirmed that GPR120 gene was knockout in GPR120 KO mice (Fig. 1B). Although no statistically significant difference was observed in cortical volume (Fig. 1C), a statistically significant hippocampus-specific decline in tissue volume was observed in GPR120 KO mice (Fig. 1D). To investigate detailed hippocampal structure, we counted pyramidal neurons in Nissl-stained sections of CA1, CA2, and CA3. The number of Nissl positive cells in the hippocampus of GPR120 KO mice was significantly decreased compared with that of WT mice (Fig. 1E), particularly in CA1 (Additional file 1: Fig. S1A, B) and CA3 (Additional file 1: Fig. S1A, D), but not in CA2 (Additional file 1: Fig. S1A, C). Neither GPR120 KO nor WT mice exhibited any sign of neuronal death in the hippocampus, as determined by FJC staining, a specific staining for degenerative neurons (Additional file 1: Fig. S1E). Although numerous FJC-positive neurons were detected in the WT hippocampus after KA-induced excitotoxicity, they were hardly detectable in the GPR120 KO hippocampus (Additional file 1: Fig. S1E). Therefore, reduced hippocampal volume in GPR120 KO mice was considered to be independent from neuronal death.

To evaluate neurogenesis in the hippocampus, we examined the level of expression of DCX and Ki67, which are neurogenesis markers [28]. The decreased level of hippocampal DCX protein (Fig. 1F, G) and DCX positive cells (Fig. 1H, I) revealed reduced neurogenesis in the GPR120 KO hippocampus. The dentate gyrus is the hippocampal region, where life-long neurogenesis occurs [29]. The frequency of Ki67-positive cells in the dentate gyrus of GPR120 KO mice was markedly lower than that in WT mice (Fig. 1J, K). SOD2 and 14-3-3ς expressions also play an important role in neurogenesis [30, 31]. The expression level of both SOD2 and 14–3-3 ς was significantly reduced in the GPR120 KO hippocampus (Fig. 1F, L and M) (Additional file 1: Fig. S1F, G). Nrf2 is a transcription factor that induces SOD2 gene expression [32]. The expression of Nrf2 protein was reduced in the GPR120 KO hippocampus (Additional file 1: Fig. S1H).

We examined the level of synaptic protein expression and conducted behavioral tests on working memory and spatial learning. The expression of the presynaptic protein synaptophysin (Fig. 1F, N) and the postsynaptic protein PSD95 (Fig. 1F, O) were reduced in the GPR120 KO hippocampus. Y-maze spontaneous alternation test results indicated that GPR120 KO mice had impaired working memory (Fig. 1P). There was no difference between GPR120 KO (21.4 ± 2.6) and WT (23.3 ± 1.92) mice in total number of arm entries. Two sets of Morris water maze trial were used to evaluate spatial reference memory-place trials (submerged platform) and probe trials (removed platform). In the place trials, the escape latency time of GPR120 KO mice was longer than that of WT mice (Fig. 1Q). Furthermore, the cross-platform time of GPR120 KO mice was shorter than that of WT mice in the probe test (Fig. 1R). There was no difference between GPR120 KO (21.3 ± 0.96 cm/s) and WT (22.6 ± 1.47 cm/s) mice in swimming velocity. These results indicated the presence of cognitive decline in GPR120 KO mice.

### Microglial activation and PGD_2_ production observed in the hippocampus of WT and GPR120 KO mice

**Fig. 2:**
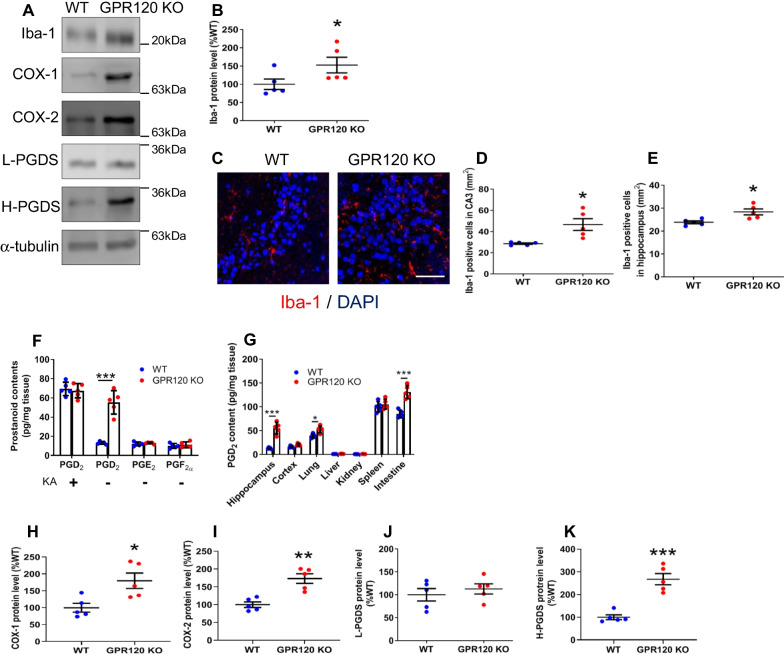
Microglial activation and PGD_2_ production observed in the hippocampus of WT and GPR120 KO mice. The protein level determined by western blot analysis (**A**). The level of Iba-1 protein in the hippocampus (**B**). The immunofluorescence of Iba-1 (**C**–**E**) and Iba-1 positive cell counts in the CA3 (D) and hippocampus (**E**). Scale bar = 50 μm. PGD_2_, PGE_2_, and PGF_2α_ contents (**F**) in the hippocampus. PGD_2_ contents in each tissue (**G**). The level of COX-1 (**H**), COX-2 (**I**), L-PGDS (**J**), and H-PGDS (**K**) protein in the hippocampus. Data are means ± SEM, *n* = 5 per group. Statistical analysis was performed using unpaired Student's *t* test (*p < 0.05; **p < 0.01; ***p < 0.001 vs. WT)

We examined microglial activation in the hippocampus by measuring the level of Iba-1 expression. There was an upregulation of Iba-1 mRNA (Additional file 2: Fig. S2A) and protein (Fig. 2A, B) in the hippocampus of GPR120 KO mice, corresponding to the increase in the number of Iba-1-positive microglia in the CA1 (Additional file 2: Fig. S2B, C), CA3 (Fig. 2C, D), and hippocampus (Fig. 2E) of GPR120 KO mice. In addition, mRNA expression of pro-inflammatory cytokines (TNFα, IL-1β, and IL-6) in the GPR120 KO hippocampus was upregulated (Additional file 2: Fig. S2D–F).

Previously we demonstrated that a surge in PGD_2_ production enhanced persistent microglial activation in the hippocampus after KA-induced excitotoxicity [9, 10]. In the present study, we found an increase in hippocampal PGD_2_ production in intact GPR120 KO mice without the administration of KA, to a level that was almost comparable to the level observed in the hippocampus of WT mice that received KA (Fig. 2F). PGE_2_ and PGF_2α_ productions remained unchanged in the GPR120 KO hippocampus (Fig. 2F). The PGD_2_ contents were slightly elevated in various organs of GPR120 KO mice compared with WT mice, but the PGD_2_ content was not elevated in the cortex (Fig. 2G). To investigate which enzymes were responsible for PGD_2_ production in the GPR120 KO hippocampus, we measured the level of protein expression of PGD_2_ synthesis enzymes, COX-1, COX-2, L-PGDS, and H-PGDS (Fig. 2A, H–K). The level of COX-1, COX-2, and H-PGDS protein was higher in the GPR120 KO hippocampus than in the WT hippocampus (Fig. 2A, H, I and K). The expression level of genes encoding these enzymes was also higher in the GPR120 KO hippocampus than in the WT hippocampus (Additional file 2: Fig. S2G–J). Especially notable were the upregulation of H-PGDS gene and protein expressions (Fig. 2A, K) (Additional file 2: Fig. S2J).

### Inhibition of PGD_2_ suppressed microglial activation and prevented the neurodegeneration in the GPR120 KO mice

**Fig. 3:**
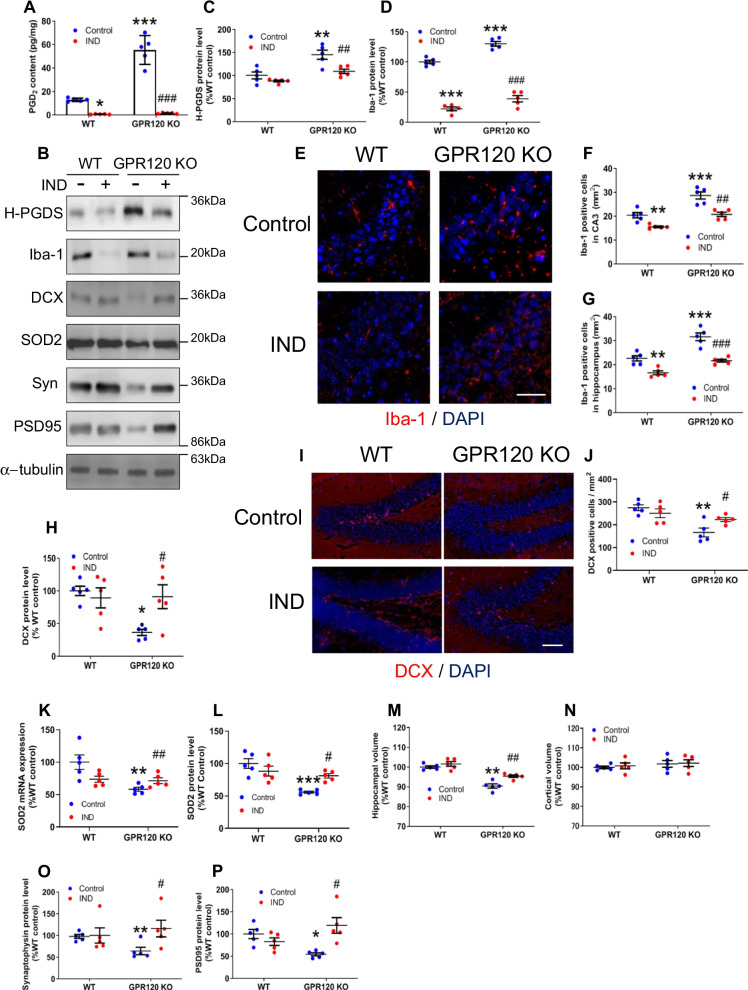
Inhibition of PGD_2_ suppressed microglial activation and prevented the neurodegeneration in the GPR120 KO mice. WT and GPR120 KO mice were administered indomethacin (IND) for 11 weeks. PGD_2_ contents in the hippocampus (**A**). The protein level determined by western blot analysis (**B**). The level of H-PGDS (**C**) and Iba-1 (**D**) protein in the hippocampus. Immunofluorescence staining of Iba-1 (**E**) and Iba-1-positive cell counts in the CA3 (**F**) and hippocampus (**G**). Scale bar = 50 μm. The level of DCX protein in the hippocampus (**H**). The immunofluorescence of DCX (**I**) and DCX-positive cell counts in the dentate gyrus (**J**). Scale bar = 80 μm. The level of SOD2 mRNA (**K**) and protein (**L**) expression in the hippocampus. Hippocampal (**M**) and cortical (**N**) volume of WT and GPR120 KO mice. The level of synaptophysin (Syn) (**O**) and PSD95 (**P**) protein in the hippocampus. Data are mean ± SEM, *n* = 5 per group. Statistical analysis was performed using two-way ANOVA followed by post-hoc Tukey test (*p < 0.05; **p < 0.01; ***p < 0.001 vs. WT control, ^#^*p* < 0.05; ^##^*p* < 0.01; ^###^*p* < 0.001 vs. GPR120 KO control)

To elucidate whether elevated PGD_2_ production was associated with hippocampal neurodegeneration in GPR120 KO mice, we treated mice with IND to suppress PGD_2_ production via inhibition of COXs. Similar to our previous results [8], IND treatment almost completely inhibited hippocampal PGD_2_ production in both groups of mice (Fig. 3A). The level of H-PGDS protein in the GPR120 KO hippocampus decreased upon inhibition of PGD_2_ production (Fig. 3B, C). The reduction in PGD_2_ production also reduced the level of Iba-1 protein (Fig. 3B, D) and Iba-1 positive microglia in the hippocampus of GPR120 KO and WT mice (Fig. 3E, F, and G) (Additional file 3: Fig. S3A, B). mRNA expression of TNFα, IL-1β, and IL-6 was ameliorated by IND treatment (Additional file 3: Fig. S3C–E). Moreover, inhibition of PGD_2_ production increased DCX protein expression (Fig. 3B, H), DCX-positive cells (Fig. 3I, J), and expression of SOD2 (Fig. 3B, K, and L), 14-3-3ς (Additional file 3: Fig. S3F, G), and Nrf2 (Additional file 3: Fig. S3H) in the hippocampus of GPR120 KO mice. Furthermore, inhibition of PGD_2_ production significantly attenuated the reduction of hippocampal volume in GPR120 KO mice (Fig. 3M), but did not affect cortical volume in mice of either group (Fig. 3N). The synaptic proteins, synaptophysin and PSD95, were increased by inhibition of PGD_2_ production in GPR120 KO mice (Fig. 3B, O, and P).

### Expression of PGD_2_ synthesis enzymes and an autocrine manner of microglial activation by PGD_2_

**Fig. 4:**
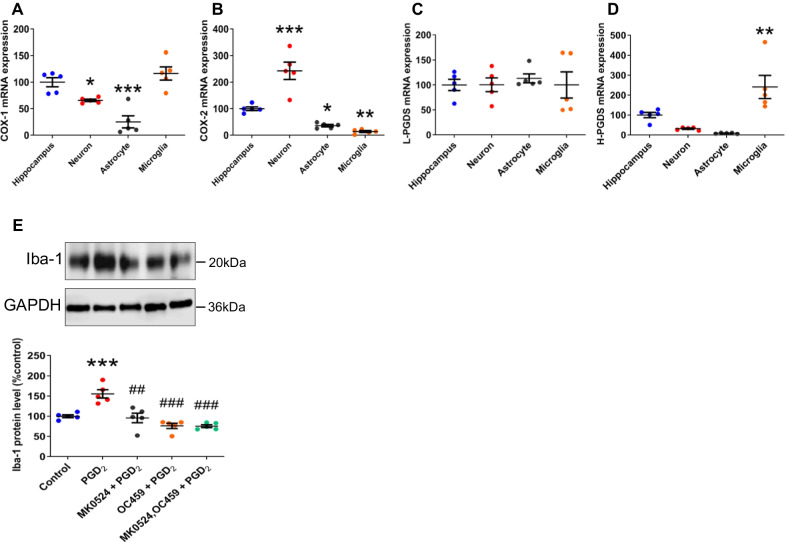
Expression of PGD_2_ synthesis enzymes and an autocrine manner of microglial activation by PGD_2_. Expression of COX-1 (**A**), COX-2 (**B**) L-PGDS (**C**), and H-PGDS (**D**) mRNA in the WT hippocampal tissue and primary cultures of Neurons, Astrocytes, and Microglia. Data are mean ± SEM, *n* = 5 per group. Statistical analysis was performed using one-way ANOVA followed by post-hoc Newman–Keuls test (*p < 0.05; **p < 0.01; ***p < 0.001 vs. Hippocampus). The protein level of Iba-1 in the primary microglia stimulated with PGD_2_ 1 h after addition of DP antagonists (**E**). Data are mean ± SEM, *n* = 5 per group. Statistical analysis was performed using one-way ANOVA followed by post-hoc Newman–Keuls test (***p < 0.001 vs. Control, ^##^*p* < 0.01; ^###^*p* < 0.001 vs. PGD_2_)

The primary cells derived from each isolation method were determined by PCR using NeuN, GFAP, and Iba-1 primers (Additional file 4: Fig. S4A). To investigate which cell types could produce PGD_2_ in the hippocampus, we examined the level of gene expression of PGD_2_ synthesis enzymes in primary neurons, astrocytes, and microglia (Fig. 4A–D). COX-1 and H-PGDS were mainly expressed in microglia (Fig. 4A, D), COX-2 was mainly expressed in neurons (Fig. 4B), and L-PGDS exhibited a similar level of expression in all three cell types (Fig. 4C). These results suggested that microglia are major producers of PGD_2_, which is in agreement with previous report [33]. We also demonstrated that PGD_2_ addition increased the level of microglial Iba-1, which was attenuated by DP1 and DP2 antagonists (Fig. 4E), suggesting that microglial activation via PGD_2_ was in an autocrine manner.

### Peripheral and intracerebral GLP-1 level and liraglutide reduced microglial PGD_2_ production

**Fig. 5:**
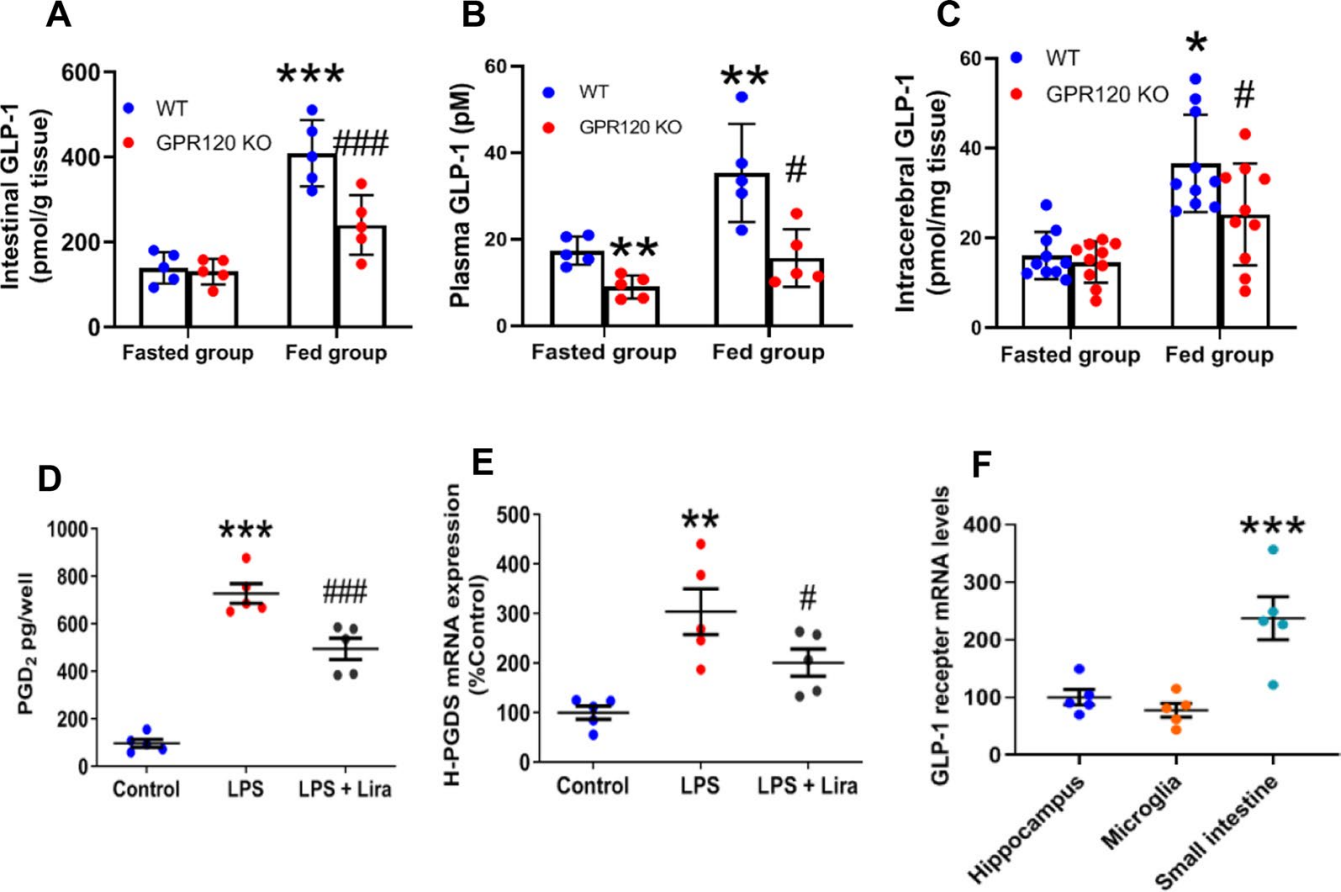
GPR120 mRNA expression profiles, tissue level of GLP-1, and Liraglutide reduced microglial PGD_2_ production. GLP-1 contents in intestine (**A**), plasma (**B**), and whole brain (**C**) of WT and GPR120 KO mice. Data are presented as the mean ± SEM, *n* = 5 or 10 per group. Statistical analysis was performed using two-way ANOVA followed by post-hoc Tukey test (**p < 0.01; ***p < 0.001 vs. WT Fasted group, ^#^*p* < 0.05; ^##^*p* < 0.01 vs. WT Fed group). PGD_2_ contents in the primary microglia stimulated with LPS for 1 h with liraglutide (Lira) addition (**D**). The level of H-PGDS mRNA expression relative to PGK1 in primary microglia (**E**). Data are mean ± SEM, *n* = 5 per group. Statistical analysis was performed using one-way ANOVA followed by post-hoc Newman–Keuls test (**p < 0.01; ***p < 0.001 vs. Control, ^#^*p* < 0.05; ^##^*p* < 0.01 vs. LPS). The level of GLP-1 receptor mRNA expression relative to PGK1 in hippocampus, microglia, and small intestine (**F**). Data are mean ± SEM, *n* = 5 per group. Statistical analysis was performed using one-way ANOVA followed by post-hoc Newman–Keuls test (**p < 0.01 vs. Hippocampus)

To investigate the peripheral and intracerebral level of GLP-1, an incretin that is secreted via PUFA/GPR120 signaling [11], we measured the level of intestinal, plasma, and intracerebral GLP-1 under fasting and fed states. Although we observed dietary elevation of intestine, plasma, and intracerebral GLP-1 contents in WT mice, no such elevation was observed in GPR120 KO mice (Fig. 5A–C). The intestinal, plasma, and intracerebral GLP-1 level in GPR120 KO mice was lower than that in WT mice during fed states (Fig. 5A–C). To investigate whether GLP-1 bioactivity directly affected microglial PGD_2_ production, we added a GLP-1 analogue, liraglutide, to primary microglial cell cultures. PGD_2_ production and H-PGDS mRNA expression increased in the LPS-stimulated primary microglia and decreased following addition of liraglutide (Fig. 5D, E). The expression of GLP-1 receptor mRNA was detected not only in the small intestine, but also in hippocampal tissue and primary microglia (Fig. 5F). These data indicated that GLP-1 bioactivity directly reduced PGD_2_ production in microglia.

### Liraglutide treatment reduced PGD_2_-microglia-provoked neuroinflammation and further neurodegeneration in GPR120 KO mice

**Fig. 6:**
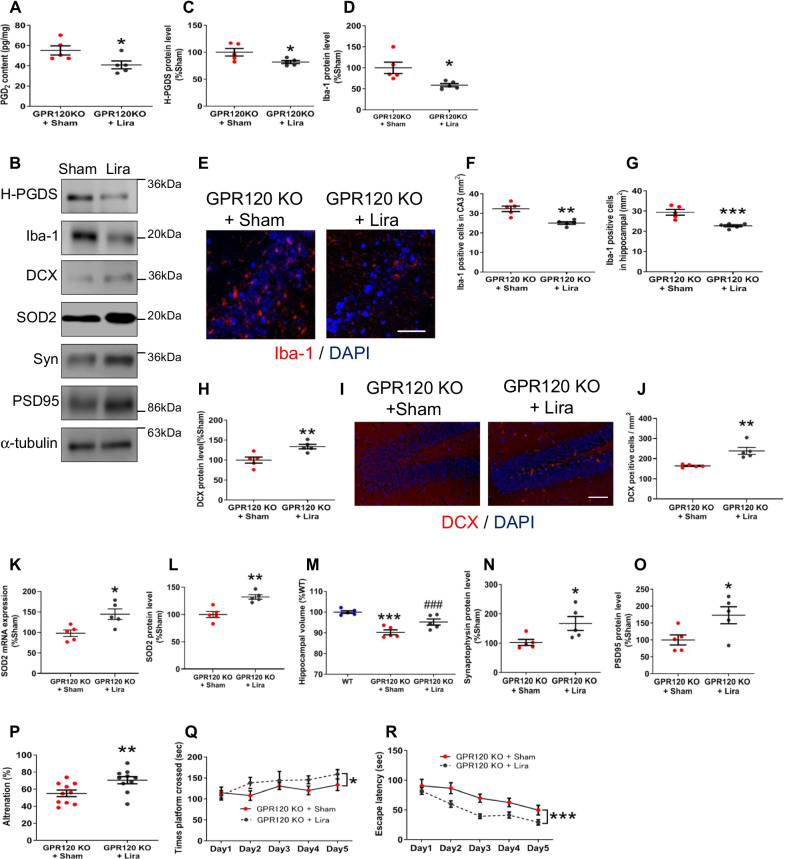
Liraglutide treatment reduced PGD_2_-microglia-provoked neuroinflammation and further neurodegeneration in GPR120 KO mice. GPR120 KO mice were administrated liraglutide (Lira) peripherally for 11 weeks. PGD_2_ contents in the hippocampus (**A**). The protein level determined by western blot analysis (**B**). The level of H-PGDS (**C**) and Iba-1 protein (**D**) in the hippocampus. The immunofluorescence of Iba-1 (**E**) and Iba-1-positive cell counts in the CA3 (**F**) and hippocampus (**G**). Scale bar = 50 μm. The level of DCX protein in the hippocampus (**H**). The immunofluorescence of DCX (**I**) and DCX-positive cell counts in the dentate gyrus (**J**). Scale bar = 80 μm. The level of SOD2 mRNA (**K**) and protein (**L**) expression. Data are presented as the mean ± SEM, *n* = 5 per group. Statistical analysis was performed using a student’s *t* test (*p < 0.05; **p < 0.01 vs. GPR120 KO + Sham). Hippocampal volume of WT and GPR120 KO mice, and Liraglutide-treated GPR120 KO mice (**M**). Statistical analysis was performed using one-way ANOVA followed by Newman–Keuls post-hoc test (***p < 0.001 vs. WT, ^#^*p* < 0.05 vs. GPR120 KO + Sham). The level of synaptophysin (Syn) (**N**) and PSD95 (**O**) protein in the hippocampus. Learning and memory performance were evaluated using the Y-maze (**P**). Data are presented as the mean ± SEM, *n* = 5 per group. Statistical analysis was performed using a student’s *t* test (*p < 0.05 vs. GPR120 KO + Sham). Morris water maze test: Escape latency (**Q**) and Time platform crossed (**R**). Data are mean ± SEM, *n* = 10 per group. Statistical analysis was performed using two-way ANOVA followed by post-hoc Tukey test (*p < 0.01; ***p < 0.001 vs. WT)

To elucidate the relationship between the level of peripheral GLP-1 and neurological phenotypes, we attempted to potentiate peripheral GLP-1 bioactivity in GPR120 KO mice. Oral administration of SPM, an inhibitor of dipeptidyl peptidase-4 (DPP-4), an enzyme that degrades GLP-1, reduced PGD_2_ production in the GPR120 KO hippocampus (Additional file 5: Fig. S5A). Peritoneal treatment with liraglutide reduced PGD_2_ production (Fig. 6A) and the level of H-PGDS protein (Fig. 6B, C) in GPR120 KO hippocampus. Iba-1 gene (Additional file 5: Fig. S5B) and protein level (Fig. 6B, D) and Iba-1 positive microglia (Fig. 6E, F, and G) (Additional file 5: Fig. S5C, D) were reduced by liraglutide. mRNA expression of TNFα and IL-1β was also ameliorated by liraglutide (Additional file 5: Fig. S5E, F), except for IL-6 (Additional file 5: Fig. S5G). Moreover, peritoneal treatment with liraglutide increased the hippocampal DCX protein expression (Fig. 6B, H), DCX-positive cells (Fig. 6I, J), expression of SOD2 (Fig. 6B, K, and L), 14-3-3ς (Additional file 5: Fig. S5H, I), Nrf2 (Additional file 5: Fig. S5J), synaptophysin (Fig. 6B, N), and PSD95 (Fig. 6B, O), and attenuated the reduction in hippocampal volume (Fig. 6M) in GPR120 KO mice. Furthermore, treatment with liraglutide improved behavioral outcomes as measured in the Y-maze (Fig. 6P) and the Morris water maze tests (Fig. 6Q, R), indicating that cognitive decline in GPR120 KO mice was ameliorated by this treatment.

## Discussion

**Fig. 7:**
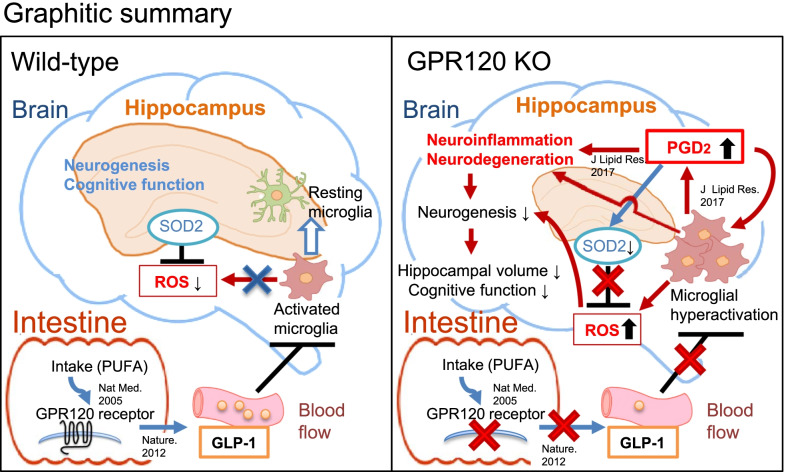
Dysfunction of peripheral GPR120 caused PGD_2_-microglia-provoked neuroinflammation and neurodegeneration in the hippocampus. Peripheral GLP-1 by intestinal GPR120 stimulation remotely contributed to hippocampal homeostasis via suppression of PGD_2_-microglia-provoked neuroinflammation in WT mice. However, insufficient GLP-1 bioactivity caused by GPR120 dysfunction induced PGD_2_-microglia-provoked neuroinflammation and neurodegeneration in GPR120 KO mice

In the current study, to reveal the relationship between PGD_2_-microglia-provoked neuroinflammation and intestinal PUFA/GPR120 signaling, we performed neurological analysis of GPR120 KO mice. We revealed that GPR120 KO mice exhibited constant hippocampal neuroinflammation, as characterized by increased PGD_2_ production and microglial activation, and various symptoms of neurodegeneration: declines in hippocampal volume, hippocampal cell number, neurogenesis, and cognitive function. We also demonstrated that inhibition of PGD_2_ production attenuated PGD_2_-microglia-provoked neuroinflammation and declines in neurogenesis, hippocampal volume, and synaptic protein level in the hippocampus of GPR120 KO mice. Furthermore, the potentiation of peripheral GLP-1 bioactivity in GPR120 KO mice by liraglutide prevented PGD_2_-microglia-provoked neuroinflammation and further neurodegeneration. These results indicated that PGD_2_-microglia-provoked neuroinflammation triggered the neurodegeneration observed in GPR120 KO mice, which could be suppressed by an increase in peripheral GLP-1 bioactivity. In addition, GPR120 mRNA was expressed in intestinal tissues, but we did not detect it in brain tissues (the whole brain, the cortex, or the hippocampus). These results indicate that neuroinflammation and neurodegeneration observed in GPR120 KO mice are probably caused by defects in intestinal GPR120 function. Therefore, we focused on the incretin, GLP-1, as it is secreted via intestinal PUFA/GPR120 signaling [11], crosses BBB [17], and increases neuronal activities [19]. Taken together with the reduced the level of GLP-1 in the intestine and plasma of GPR120 KO mice, their neurological phenotypes were caused by a decline of intracerebral GLP-1, which was caused in turn by insufficient GLP-1 secretion from GPR120 signaling-defective intestine and low entry of GLP-1 into the brain. In the GPR120 KO hippocampus, increased PGD_2_ production downregulated SOD2 expression, which would fail to scavenge reactive oxygen species (ROS), a leading cause of reduced neurogenesis. Thus, intestinal GLP-1 bioactivity by GPR120 stimulation may remotely contribute to hippocampal homeostasis via suppression of PGD_2_-microglia-provoked neuroinflammation (Fig. 7). Microglia produce pro-inflammatory cytokines (TNFα, IL-1β, and IL-6) as part of their neuroinflammatory response [34]. The mRNA expression of these cytokines in the GPR120 KO hippocampus were upregulated (Additional file 2: Fig. S2D–F) and their activation was ameliorated by IND (Additional file 3: Fig. S3C–E) and liraglutide (Additional file 5: Fig. S5E–G) treatments. These results suggest that microglia in GPR120 KO hippocampus were functionally activated by PGD_2_ and their activities could be suppressed by intestinal GLP-1 bioactivity.

In the current study, we used 16-week-old GPR120 KO mice for all experiments. This is because while GPR120 KO mice fed a high-fat diet are susceptible to obesity, when fed a normal diet, these mice’s physical parameters, including body weight, food intake, energy expenditure, locomotor activity, and plasma adiponectin level do not significantly differ from those of WT mice [13]. In addition, body weight, total number of entries in Y-maze test, and swimming velocity in the Morris water maze test were not significantly different between WT and GPR120 KO mice in this study. Therefore, any neurological phenotypes observed in GPR120 KO mice were not considered to be induced by systemic inflammation associated with obesity.

We considered that PGD_2_-microglia-provoked neuroinflammation was the main cause of neurodegeneration observed in GPR120 KO mice. Our in vitro experiments with primary microglial cell cultures revealed that H-PGDS expression increased PGD_2_ production and microglial Iba-1 expression, which could be blocked by DP1- and DP2-antagonists. These data suggest that microglia were activated by PGD_2_ in an autocrine manner. An upregulation of hippocampal PGD_2_ in GPR120 KO mice was observed even without KA-administration, at a level that was almost equal to that in the hippocampus of KA-administrated WT mice. Constant PGD_2_-induced microglial activation results in neuroinflammation in the hippocampus of GPR120 KO mice without external stimulation. Previously we reported a surge in PGD_2_ production in the hippocampus of KA-administrated WT mice [8], which led to PGD_2_-enhanced persistent microglial activation [9, 10]. In this study, we showed that microglia expressed H-PGDS and microglia played a major role in PGD_2_ production. These experimental results suggest that hippocampal PGD_2_ produced by microglia causes microglial activation observed in the GPR120 KO hippocampus. Excessive microglial activation plays a pivotal role in neuroinflammation and causes neurodegeneration by inhibiting neurogenesis via the secretion of ROS [35–37]. Previous study reported that SOD2 KO mice exhibited reduced neurogenesis [38], indicating that scavenging of ROS by SOD2 plays an important role in neurogenesis. In our results, microglial PGD_2_ production downregulated SOD2 expression in the GPR120 KO hippocampus. In addition, we found that Nrf2 protein expression was downregulated in the GPR120 KO hippocampus (Additional file 1: Fig. S1H), but could be increased by IND (Additional file 3: Fig. S3H) or liraglutide (Additional file 5: Fig. S5J) treatments. Nrf2 is a transcription factor that induces SOD2 gene expression when bound to the SOD2 promoter [32]. These results suggest that PGD_2_-microglia-provoked neuroinflammation may inhibit neurogenesis by downregulating the Nrf2/SOD2 mediated anti-oxidant pathway. Although 15-deoxy-Δ^12, 14^-PGJ_2_ (15d-PGJ_2_), a non-enzymatically converted metabolite of PGD_2_, has been reported to activate Nrf2 [39, 40], the amount of 15d-PGJ_2_ in KA-stimulated hippocampus was almost undetectable in our previous lipidomics analysis [8]. Although detailed mechanisms are unknown, PGD_2_ may downregulate Nrf2 expression in this neuroinflammatory response. In addition to SOD2, 14-3-3ς protein also affects neurogenesis through regulation of neuronal differentiation into neurons [31] and its expressions was reduced by microglial PGD_2_ production. Thus, microglial PGD_2_ production inhibited neurogenesis via suppression of SOD2 and 14-3-3ς expressions, which is in agreement with a study of Alzheimer’s disease mouse model [41]. Furthermore, in vitro cell culture studies have demonstrated that addition of PGD_2_ enhances ROS generation [42, 43]. Therefore, PGD_2_-microglia-provoked neuroinflammation probably lead to the neurodegeneration in GPR120 KO hippocampus by inhibiting neurogenesis. We observed this neuroinflammatory pathway specifically in the hippocampus. One possible reason may be the potential ability of the hippocampus to produce PGD_2_. We previously reported that KA-induced elevation of PGD_2_ production was observed in the hippocampus, and not in the cortex [8], suggesting that this pathway was activated specifically in the hippocampus, probably due to its higher ability to produce PGD_2_.

We observed declines in neurogenesis, hippocampal volume, and cognitive function in GPR120 KO mice in the present study. Neurogenesis is an important contributor to hippocampal volume and structure [44, 45]. Reduced hippocampal volume is associated with cognitive decline in neuronal disorders [46–48]. In addition, it is well known that cognitive decline in Alzheimer’s disease and type 2 diabetes mellitus are correlated with a decrease in hippocampal volume [49, 50]. A genome-wide association analysis conducted as part of the Alzheimer’s disease Neuroimaging Initiative revealed a significant relationship between neurogenesis and hippocampal volume in humans [45]. Further, the main GLP-1 degrading enzyme, DPP-4, is associated with hippocampal volume, suggesting that an insufficient GLP-1 level may also be related to hippocampal volume [45]. These observations suggest that insufficient GLP-1 level may contribute to neurodegeneration in neuronal diseases exhibiting cognitive impairment.

In our results, GPR120 KO mice showed a reduced level of intestinal, plasma, and intracerebral GLP-1, suggested that an insufficiency of intestinal GLP-1 secretion could be a cause of decline in intracerebral GLP-1 level. Supporting this concept, GLP-1 is known to be secreted from enteroendocrine L cells in the intestinal epithelium and can act on other organs, such as the pancreas, via systemic circulation [51]. Since SPM, an inhibitor of DPP-4, does not cross the BBB [52], it can inhibit the degradation of peripheral GLP-1, but not of GLP-1 in the brain. The fact that oral administration of SPM enhances GLP-1 level in the brain [53], indicates that elevation of peripheral GLP-1 level can elevate the intracerebral GLP-1 via the bloodstream. In the present study, we showed that oral administration of SPM reduced hippocampal PGD_2_ production in GPR120 KO mice (Additional file 5: Fig. S5A). In addition, we demonstrated that GLP-1 receptor was expressed in microglia, GLP-1 bioactivity reduced microglial H-PGDS expression and PGD_2_ production, and microglia were the main cells to produce PGD_2_ in the brain. Previous studies have reported that PGD_2_ synthesis by H-PGDS is ROS-dependent (i.e., Ros directly upregulates H-PGDS activity) [54, 55] and GLP-1 reduces the accumulation of intracellular ROS by increasing the expression of antioxidant enzymes in BV-2 microglia [56]. Thus, a candidate mechanism for reduction of microglial H-PGDS activity by GLP-1 is suppression of ROS production via the GLP-1 receptor. Taken together, these observations suggest that intestinal GLP-1, secreted by intestinal GPR120 stimulation, and transported via the bloodstream, acts remotely on microglia by reducing microglial PGD_2_ production in the hippocampus.

In this study, GPR120 mRNA was abundantly expressed in intestinal tissues, but we did not detect it in the hippocampus, the cortex, or the whole brain. Our data are in agreement with studies demonstrating the tissue specific expression of GPR120 [11, 57]. We considered that intestinal GPR120 probably makes a large contribution to suppress PGD_2_-microglia-provoked neuroinflammation and neurodegeneration in the hippocampus. Some studies have reported GPR120 expression in the hypothalamus and pituitary [58–60], but the reported level of expression was much lower than that in intestinal tissues. We found a remote effect of GPR120 using GPR120 KO mice, but further research is needed to link this effect to human physiological and pathological conditions.

In this study, dietary elevation of GLP-1 level was not observed in GPR120 KO mice. We have previously reported that GPR120 senses and responds to several n-3 PUFAs [11, 12], such as ALA, EPA, and DHA. Although it is well known that dietary ALA is converted to EPA and DHA, ALA itself exerts a neuroprotective effect against excitotoxicity [61] and can be used in the synthesis of palmitic acid and cholesterol, precursors in myelin synthesis [62–64]. Furthermore, EPA and DHA are essential fatty acids for brain development, functions, and neuroprotection [65]. Thus, to sense and detect these key PUFAs, such as ALA, EPA, and DHA, by GPR120 might mediate an important strategy for maintaining hippocampal homeostasis.

## Conclusion

In the current study, we revealed that dysfunction of GPR120 caused PGD_2_ overproduction, persistent microglial activation, loss of neurogenesis, decreased hippocampal volume, and cognitive decline. Specifically, insufficient GLP-1 bioactivity was a result of GPR120 dysfunction, and induced PGD_2_-microglia-provoked neuroinflammation, which is a major factor of the neurodegeneration observed in GPR120 KO mice. These findings may suggest that insensitivity to dietary PUFA by dysfunction of GPR120 would raise the risk of hippocampal dysfunction. These observations may reveal the presence of a novel gut–brain interaction, in that the signaling of dietary PUFA is sensed by GPR120, converted into incretin bioactivity, and contributes to hippocampal homeostasis via suppression of PGD_2_-microglia-provoked neuroinflammation. Furthermore, our results illustrated that potentiation of GLP-1 bioactivity suppressed this neuroinflammatory pathway, indicating a potentially novel mechanism of action for incretin-based therapies, which are promising treatment options for cognitive decline in patient with Alzheimer’s disease and type 2 diabetes mellitus.

## Supplementary Information

**Additional file 1: Fig. 1.** Nissl and FJC staining and 14-3-3ς and Nrf2 expression level in the hippocampus. Nissl staining (A) and pyramidal cell counts of CA1 (B), CA2 (C) and CA3 (D). FJC staining of WT, GPR120 KO, and KA-treated WT mice hippocampus (E). The level of 14-3-3ς mRNA (F), 14-3-3ς protein (G), and Nrf2 protein (H) expression in the hippocampus. Data are presented as the mean ± SEM, *n* = 5 per group. Statistical analysis was performed using a student’s *t* test (*p < 0.05; **p < 0.01 vs. WT).

**Additional file 2: Fig. 2.** Iba-1 mRNA expression and Iba-1 positive cell counts. Gene expression level of cytokines and PGD_2_ synthesis enzymes in hippocampus. The Iba-1 mRNA expression level (A), immunofluorescence of Iba-1 (B) and Iba-1 positive cell counts in the CA1 (C). The level of TNFα (D), IL-1β (E), and IL-6 (F) mRNA expression in the hippocampus. The level of COX-1 (G), COX-2 (H), L-PGDS (I), and H-PGDS (J) mRNA relative to PGK1 in the hippocampus. Data are means ± SEM, *n* = 5 per group. Statistical analysis was performed using unpaired Student's *t* test (*p < 0.05; ***p < 0.001 vs. WT).

**Additional file 3: Fig. 3.** Inhibition of PGD_2_ suppressed microglial and cytokines activation and increased the level of 14-3-3ς and Nrf2 expression in the GPR120 KO hippocampus. The immunofluorescence of Iba-1 (A) and Iba-1 positive cell counts in the CA1 (B). The level of TNFα (C), IL-1β (D), and IL-6 (E) mRNA expression in the hippocampus. The level of 14-3-3ς mRNA (F), 14-3-3ς protein (G), and Nrf2 protein (H) expression in the hippocampus. Data are presented as the mean ± SEM, *n* = 5 per group. Statistical analysis was performed using two-way ANOVA followed by post-hoc Tukey test (*p < 0.05; **p < 0.01 vs. WT control, ^#^*p* < 0.05; ^##^*p* < 0.01, ^###^*p* < 0.001vs. GPR120 KO control).

**Additional file 4: Fig. 4.** Neuronal and glial marker expressions in primary cell cultures. PCR analysis for NeuN, GFAP, and Iba-1 in primary cultures of Neuron, Astrocyte, and Microglia (A).

**Additional file 5: Fig. 5.** Oral administration of SPM reduced hippocampal PGD_2_ production. Peripheral administration of liraglutide reduced microglial and cytokines activation and increased the level of 14-3-3ς and Nrf2 expression in the GPR120 KO hippocampus. PGD_2_ contents in the hippocampus of WT, GPR120 KO, and SPM-treated GPR120 KO mice (A). Data are presented as the mean ± SEM, *n* = 5 per group. Statistical analysis was performed using one-way ANOVA followed by Newman–Keuls post-hoc test (***p < 0.001 vs. WT, ^###^*p* < 0.001 vs. GPR120 KO + Sham). The level of Iba-1 mRNA expression in the hippocampus (B). The immunofluorescence of Iba-1 (C) and Iba-1 positive cell counts in the CA1 (D). The level of TNFα (E), IL-1β (F), and IL-6 (G) mRNA expression in the hippocampus. The level of 14-3-3ς mRNA (H), 14-3-3ς protein (I), and Nrf2 protein (J) expression in the hippocampus. Data are presented as the mean ± SEM, *n* = 5 per group. Statistical analysis was performed using a student’s *t* test (*p < 0.05; **p < 0.01 vs. Sham).

## Data Availability

The data sets used and/or analyzed during the current study are available from the corresponding author by reasonable request.

## Abbreviations

ALA: α-Linolenic acid
COX: Cyclooxygenase
DCX: Doublecortin
DHA: Docosahexaenoic acid
DPP-4: Dipeptidyl peptidase-4
EPA: Eicosapentaenoic acid
FJC: Fluoro Jade C
GFAP: Glial fibrillary acidic protein
GLP-1: Glucagon-like peptide-1
H-PGDS: Hematopoietic prostaglandin D synthase
Iba-1: Ionized calcium binding adapter molecule 1
IL: Interleukin
KA: Kainic acid
KO: Knockout
Lira: Liraglutide
L-PGDS: Lipocalin-type prostaglandin D synthase
LPS: Lipopolysaccharide
Nrf2: Nuclear factor erythroid 2-related factor 2
PG: Prostaglandin
PSD95: Postsynaptic density protein 95
PUFA: Polyunsaturated fatty acids
ROS: Reactive oxygen species
SOD: Superoxide dismutase
TNFα: Tumor necrosis factor α
WT: Wild type
