# Supplementary figures and images for: A peripheral lipid sensor GPR120 remotely contributes to suppression of PGD2-microglia-provoked neuroinflammation and neurodegeneration in the mouse hippocampus
Source: J Neuroinflammation. 2021 Dec 27;18:304. doi: 10.1186/s12974-021-02361-2 (PMC8711188; doi:10.1186/s12974-021-02361-2)

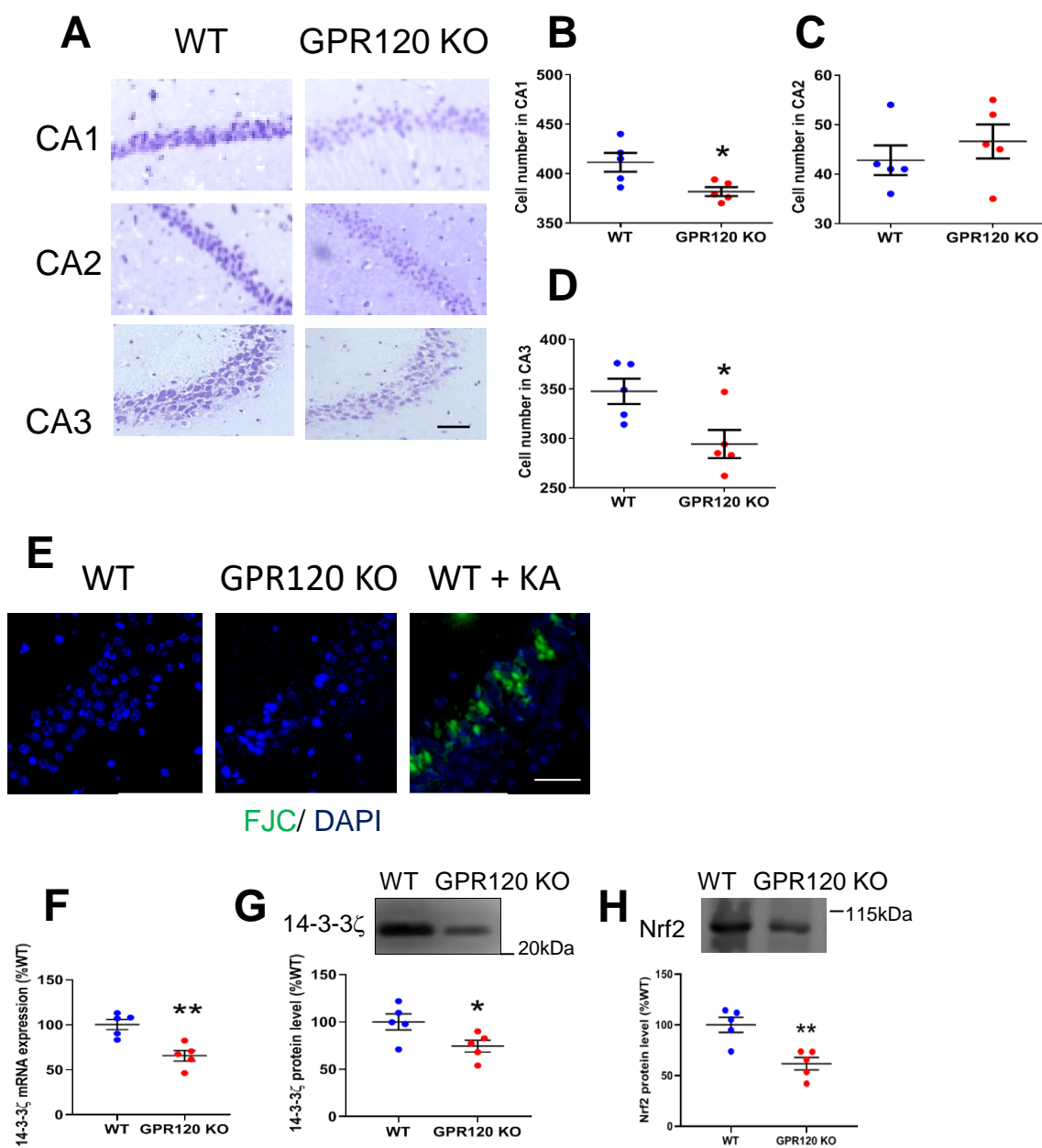

Additional Figure 1

Supplement: Supplementary file 1 — Additional file 1: Fig. 1. Nissl and FJC staining and 14-3-3ς and Nrf2 expression level in the hippocampus. Nissl staining (A) and pyramidal cell counts of CA1 (B), CA2 (C) and CA3 (D). FJC staining of WT, GPR120 KO, and KA-treated WT mice hippocampus (E). The level of 14-3-3ς mRNA (F), 14-3-3ς protein (G), and Nrf2 protein (H) expression in the hippocampus. Data are presented as the mean ± SEM, n = 5 per group. Statistical analysis was performed using a student’s t test (*p < 0.05; **p < 0.01 vs. WT). [file 12974_2021_2361_MOESM1_ESM.pdf]

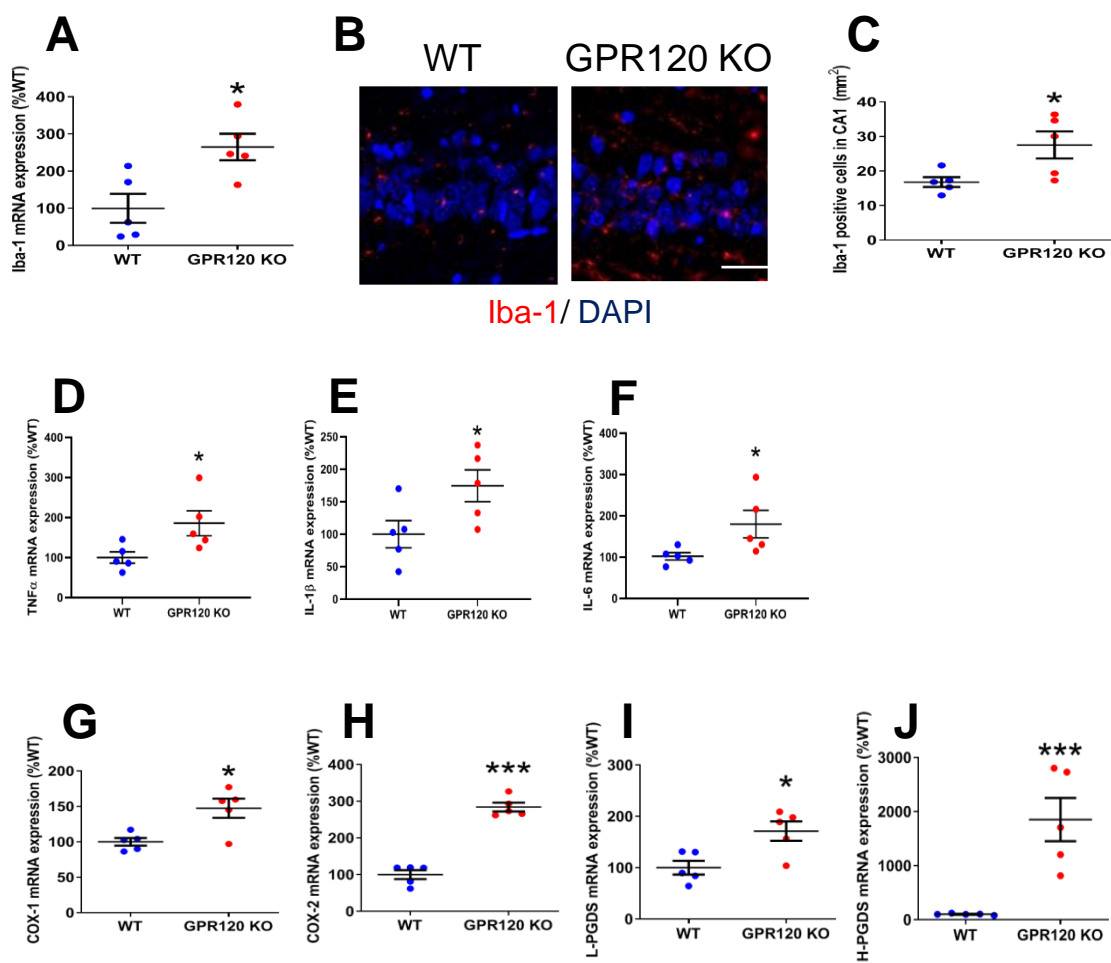

Additional Figure 2

Supplement: Supplementary file 2 — Additional file 2: Fig. 2. Iba-1 mRNA expression and Iba-1 positive cell counts. Gene expression level of cytokines and PGD2 synthesis enzymes in hippocampus. The Iba-1 mRNA expression level (A), immunofluorescence of Iba-1 (B) and Iba-1 positive cell counts in the CA1 (C). The level of TNFα (D), IL-1β (E), and IL-6 (F) mRNA expression in the hippocampus. The level of COX-1 (G), COX-2 (H), L-PGDS (I), and H-PGDS (J) mRNA relative to PGK1 in the hippocampus. Data are means ± SEM, n = 5 per group. Statistical analysis was performed using unpaired Student's t test (*p < 0.05; ***p < 0.001 vs. WT). [file 12974_2021_2361_MOESM2_ESM.pdf]

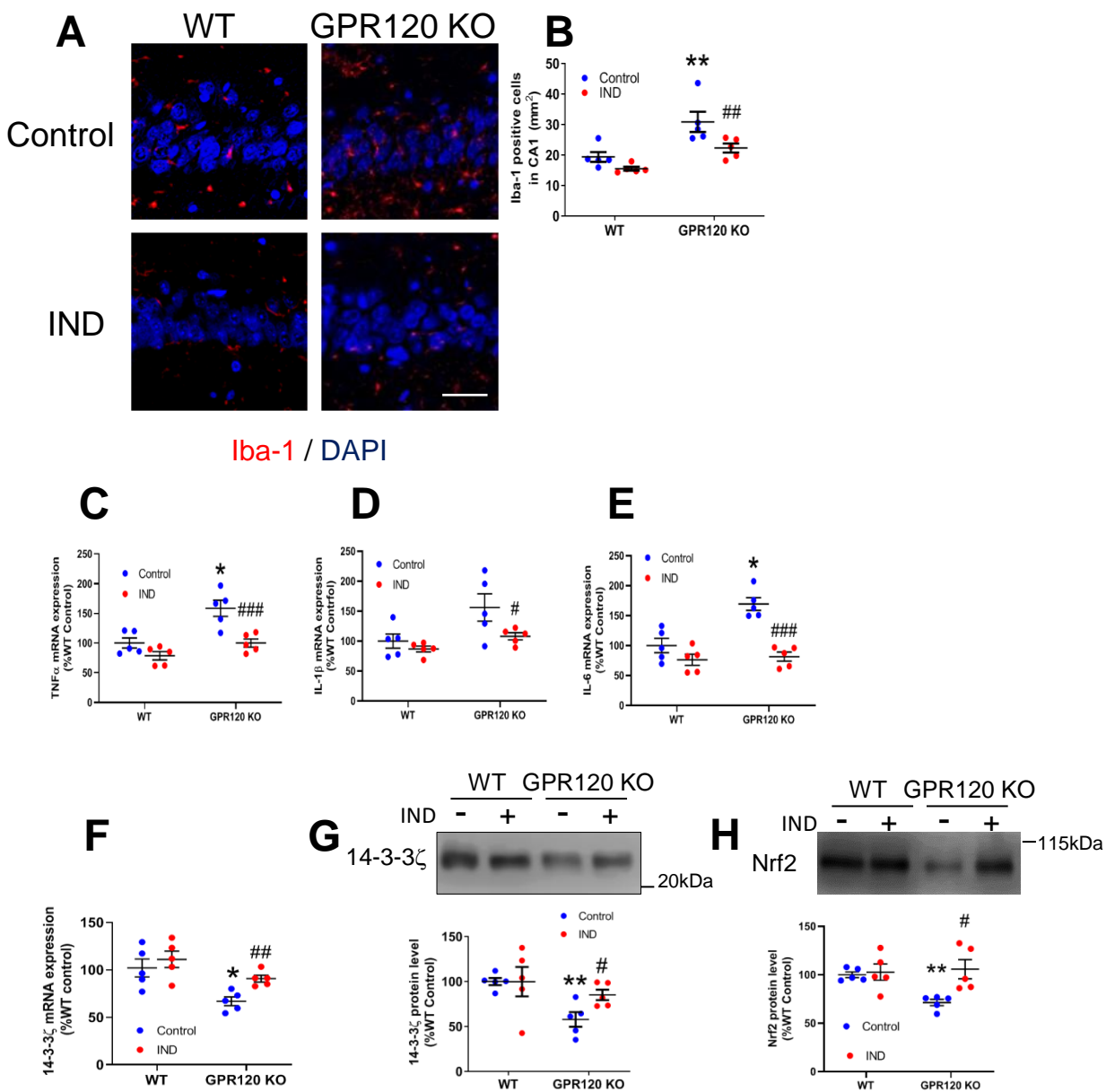

Additional Figure 3

Supplement: Supplementary file 3 — Additional file 3: Fig. 3. Inhibition of PGD2 suppressed microglial and cytokines activation and increased the level of 14-3-3ς and Nrf2 expression in the GPR120 KO hippocampus. The immunofluorescence of Iba-1 (A) and Iba-1 positive cell counts in the CA1 (B). The level of TNFα (C), IL-1β (D), and IL-6 (E) mRNA expression in the hippocampus. The level of 14-3-3ς mRNA (F), 14-3-3ς protein (G), and Nrf2 protein (H) expression in the hippocampus. Data are presented as the mean ± SEM, n = 5 per group. Statistical analysis was performed using two-way ANOVA followed by post-hoc Tukey test (*p < 0.05; **p < 0.01 vs. WT control, #p < 0.05; ##p < 0.01, ###p < 0.001vs. GPR120 KO control). [file 12974_2021_2361_MOESM3_ESM.pdf]

**A**

NeuN    GFAP    Iba-1

Neuron

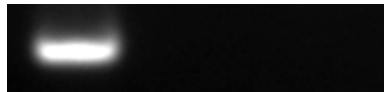

Astrocyte

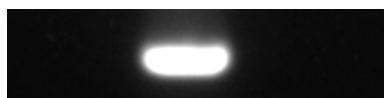

Microglia

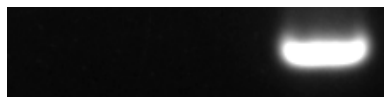

Supplement: Supplementary file 4 — Additional file 4: Fig. 4. Neuronal and glial marker expressions in primary cell cultures. PCR analysis for NeuN, GFAP, and Iba-1 in primary cultures of Neuron, Astrocyte, and Microglia (A). [file 12974_2021_2361_MOESM4_ESM.pdf]

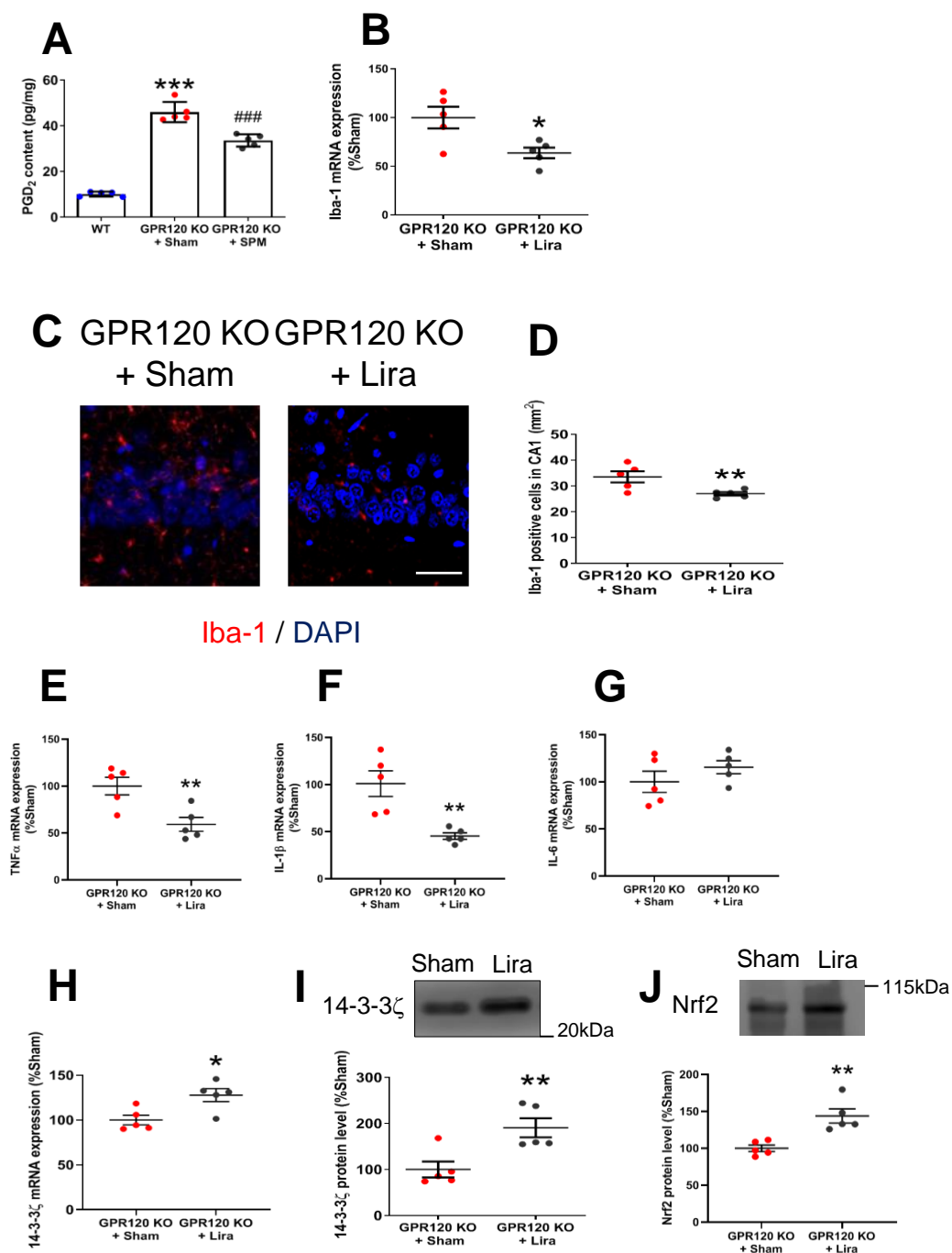

Additional Figure 5

Supplement: Supplementary file 5 — Additional file 5: Fig. 5. Oral administration of SPM reduced hippocampal PGD2 production. Peripheral administration of liraglutide reduced microglial and cytokines activation and increased the level of 14-3-3ς and Nrf2 expression in the GPR120 KO hippocampus. PGD2 contents in the hippocampus of WT, GPR120 KO, and SPM-treated GPR120 KO mice (A). Data are presented as the mean ± SEM, n = 5 per group. Statistical analysis was performed using one-way ANOVA followed by Newman–Keuls post-hoc test (***p < 0.001 vs. WT, ###p < 0.001 vs. GPR120 KO + Sham). The level of Iba-1 mRNA expression in the hippocampus (B). The immunofluorescence of Iba-1 (C) and Iba-1 positive cell counts in the CA1 (D). The level of TNFα (E), IL-1β (F), and IL-6 (G) mRNA expression in the hippocampus. The level of 14-3-3ς mRNA (H), 14-3-3ς protein (I), and Nrf2 protein (J) expression in the hippocampus. Data are presented as the mean ± SEM, n = 5 per group. Statistical analysis was performed using a student’s t test (*p < 0.05; **p < 0.01 vs. Sham). [file 12974_2021_2361_MOESM5_ESM.pdf]
